# Disclosure and health-related outcomes among children living with HIV and their caregivers

**DOI:** 10.1186/s12981-021-00337-z

**Published:** 2021-04-20

**Authors:** Margaret Amankwah-Poku, Delight Abla Klutsey, Kwaku Oppong Asante

**Affiliations:** 1 Department of Psychology, University of Ghana, Legon, Accra, Ghana; 2 Department of Psychology, University of the Free State, Bloemfontein, South Africa

**Keywords:** Children Living with HIV, Disclosure, Medication Adherence, Caregivers, Psychological health, HIV/AIDS

## Abstract

**Background:**

The prevalence of disclosure of status to children living with the Human Immunodeficiency Virus (HIV) is low in most sub-Saharan African countries, leading to poor compliance and adverse psychological outcomes in these children. This study examined the influence of disclosure on health outcomes in children living with HIV and their caregivers.

**Methods:**

Using a cross-sectional design, 155 HIV-positive children between age 6–15 years and their caregivers were administered standardized questionnaires measuring adherence to medication, children's psychological well-being, caregiver burden, and caregivers’ psychological health.

**Results:**

Results indicated that only 33.5% of the children sampled knew their status. Disclosure of HIV status was significantly related to medication adherence, psychological wellbeing, the burden of caregiving, and the length of the disclosure. A child’s age and level of education were the only demographic variables that significantly predicted disclosure of HIV status. In a hierarchical analysis, after controlling for all demographic variables medication adherence, psychological well-being and burden of caregiving were found to be significant predictors of disclosure of status in children living with HIV.

**Conclusions:**

Findings suggest the need for disclosure of status among children living with HIV for a positive impact on their medication adherence and psychological health. These findings underscore the need for the development of context-specific interventions that will guide and encourage disclosure of status by caregivers to children living with HIV.

## Background

Pediatric HIV is the infection of HIV in neonates, children, and adolescents [1, 2]. Globally, approximately 1.8 million children and adolescents below 15 years of age live with the disease, 91% of whom reside in sub-Saharan Africa [3, 1]. In Ghana, as of 2017, a total of 33,000 children were living with HIV and AIDS [2], the majority of whom are infected by mother-to-child transmission [3]. Children living with HIV face many medical and psychosocial problems that greatly contribute to the worldwide burden of HIV. Among these are poor access to antiretroviral therapy due to poverty, stigma, and low levels of HIV status disclosure [2, 4].

Disclosure of status is a pertinent issue in the management of HIV and yet a major psychosocial challenge encountered by individuals living with HIV/AIDS [8–7]. Pediatric disclosure in this study refers to a child or an adolescent’s knowledge or awareness of his/her HIV status [8]. Though disclosure has been attributed to increased adherence to antiretroviral treatment [9, 10], improved accessibility to support services, open discussions within the family setting [7, 11, 12] and improved psychological wellbeing [13, 14], it continues to be low in many low and middle-income countries, especially those in sub-Saharan Africa, including Ghana [6, 8]. Reasons for the low rates of disclosure include fear of disclosure affecting the child's psychological well-being, fear that the child might not keep the diagnosis confidential, fear of being blamed for infecting the child, stigma, and the assertion that the child may be immature to understand the meaning of the disease [15, 16]. Caregivers are therefore faced with the dilemma of whether ‘to disclose or not to disclose’ the status of their children to them. Thus, non-disclosure of HIV status has resulted in unsatisfactory health-related outcomes in children living with HIV such as poor adherence and psychological wellbeing [18–17] which can have detrimental effects that can significantly affect the health of infected children.

Disclosure also has an association with caregiver outcomes such as the burden of care and psychological health [18, 19]. In Ghana, poor health outcomes have been recorded for caregivers of children living with HIV including varying degrees of psychological distress and burden [15], similar to findings from other sub-Saharan African countries [18–20]. One reason that explains the distress experienced by caregivers is the lack of disclosure, as non-disclosure creates anxieties and tension within the family setting because there are no open discussions within the family about the child’s condition [12, 21, 22].

Although disclosure of status has benefits and non-disclosure is associated with some negative health outcomes for both children and their caregivers, most Ghanaian caregivers are unable to disclose their children’s HIV status to them. Studies have shown that disclosure rates among caregivers of children living with HIV in Ghana range from 21 to 33% [15, 23]. Despite these low levels of disclosure status, the psychosocial factors that influence disclosure of HIV status of children by their caregivers in Ghana remain unknown. This study therefore, examined factors influencing disclosure of HIV status of children by caregivers in Ghana. We specifically explored two main objectives: (1) to determine the prevalence of full disclosure of status to children living with HIV and (2) determine the predictors of full disclosure of HIV status of children by their caregivers. The outcome of this study can help develop context-specific interventions that will guide and encourage disclosure of status among caregivers of children living with HIV.

## Method

### Study context and sample

This study was conducted in the Greater Accra Region of Ghana, specifically in four main hospitals in Accra, Ghana. The Greater Accra region was selected because it is the most urbanized, cosmopolitan, and densely populated region in Ghana with dwellers and settlers from all the other regions in Ghana and beyond. Participants were included in the study if they met the following inclusion criteria: (1) a caregiver of a child between ages 6 and 15 years living with HIV; (2) the child and or adolescent attends HIV clinic in any of the 4 major referral hospitals in Accra, Ghana; and (3) the child has been enrolled on antiretroviral therapy for at least six months. Of the caregivers approached during recruitment, only five of them declined participation, as they wanted nothing to do with the study. A total of one hundred and fifty-five (155) children living with HIV/AIDS and their caregivers (age range of 22–82 years) who were purposively and conveniently recruited from the special HIV clinics at the four hospitals all completed the study. The children together with their caregivers were administered caregiver-child dyads. Participants who did not meet the inclusion criteria were excluded from participation. However, due to the sensitive nature of the study, and the stigma attached to the condition, we are unable to obtain any demographic information from such individuals (and the five caregivers who declined participation), as they did not consent to their information being obtained.

### Procedure

This study received ethics approval from the College of Humanities Ethics Committee, University of Ghana (ECH: 008/18-19), and the Greater Accra Regional Directorate of Ghana Health Services (GHS/GARHD/007/19). Permission was also obtained from all four participating hospitals. Caregivers attending antiretroviral clinics with their children were approached and the purpose, benefits, and potential risks of the study were explained to them. Caregivers who agreed to participate signed a consent form and also gave informed consent for their children to participate. The first researcher and a trained research assistant then administered a translated version of the questionnaires (in the Twi dialect) by reading out the questions and response options for participants to respond appropriately, as the majority of the caregivers could neither read nor understand English. Caregivers who could read and understand English were given the questionnaires to complete themselves. It took 15–20 min to complete each questionnaire. Caregivers did not receive any compensation for completing the questionnaires, but each child was given tokens of pens, pencils, and erasers. This process of data collection was the same for all four hospitals.

### Measures

#### Demographic questionnaire

Demographic information obtained from both caregivers and children living with HIV/AIDS included their age, sex, level of education, caregivers’ relationship with the child, disclosure of status, and the duration of disclosure if disclosure had been made.

#### HIV status disclosure

Disclosure of HIV status in children was measured using the USAID-Academic Model Providing Access to Healthcare (AMPATH) disclosure questionnaire [16]. This 15-item questionnaire with ‘yes’ and ‘no’ response options assesses disclosure of HIV status in caregivers of children living with HIV/AIDS, adherence, stigma, and depression. The first four items measuring disclosure of HIV status by caregivers of children living with HIV were used in this study. Full disclosure is said to have been made if the caregivers responded “Yes” to all four items. Non-disclosure is said to have occurred if caregivers respond “No” to any of the four items.

#### Medication adherence

The Medication Adherence Rating Scale (MARS) [24] was used to measure medication adherence in children living with HIV/AIDS. The MARS has 10 items scored with ‘yes’ or ‘no’ responses. Two items are reverse scored and total scores range from 0–10 with a higher score indicating better adherence and lower scores indicating poor adherence.

#### Well-being

The Stirling Children’s Well-being Scale [25] is a 15-items questionnaire that measures emotional and psychological wellbeing in children aged 6–15 years. It is scored on a 5 point Likert scale ranging from 1 (never) to 5 (all of the time). The questionnaire has two subscales, “positive emotional state” and “positive outlook” comprising 6 items each, and 3 social desirability indicator items. In this study, the total scale score was used instead of the subscale scores. Thus, total scores ranging from 12 to 60 with higher scores indicating higher psychological wellbeing in children.

#### Caregiver burden

The Zarit Burden Interview [26] was used to measure self-reported caregiver burden among caregivers of children living with HIV. It comprised 22 items to which caregivers responded to on a 5 point Likert scale ranging from 0 (Never) to 4 (Nearly Always). Total scores range from 0 to 88 with higher scores indicating greater caregiver distress or burden.

#### Psychological health

Caregivers’ psychological health was measured using the General Health Questionnaire- 28 (GHQ-28) [27] The questionnaire has four subscales measuring somatic symptoms, anxiety and insomnia, social dysfunction, and severe depression scored on a 4 point Likert scale ranging from 0 (not at all) to 3 (much more than usual). Total scores ranging from 0 to 84 and a score of 24 is a threshold for the presence of psychological distress.

### Statistical analysis

The Statistical Package for the Social Sciences (IBM SPSS) version 23 was used for data analyses. Descriptive statistics were used describe the data and also to determine the prevalence of HIV disclosure. The Pearson product-moment correlation coefficient was used to examine the relationship among the variables in the study. Hierarchical regression was performed to examine the relative contribution of the independent variables (i.e. medication adherence, psychological wellbeing of children with HIV, caregiver burden, and psychological wellbeing of caregivers) in predicting disclosure of HIV status (outcome variable). In the first step, the following demographic variables (child’s age, child’s level of education, caregivers’ level of education) were controlled for as these have been shown to significantly affect disclosure of status [15, 28–30]. In the second step, all the independent variables were entered into the model. This procedure was followed to ensure that those variables that were strongly associated with disclosure of HIV status would account for unique variance beyond that accounted for by the demographic variables. Statistical significance was defined as two-tailed *p*-value < 0.05.

## Results

### Sociodemographic characteristics of participants

The socio-demographic characteristics of the participants are presented in Table 1. A dyad of 155 children living with HIV and their caregivers were tested. The mean age of the caregivers was 41.60 (*SD* = 10.38) with 119 (76.8%) of them being females and majority (40.0%) of them with no formal education. The children, on the other hand, had a mean age of 9.55 (*SD* = 2.72) years with 83 (53.5%) being males. The majority of the children (66.5%) were not aware of their HIV status while 33.5% were aware of their status.

**Table 1:** Socio-demographic information of caregivers and children (N = 155)

Variables	Number	Percentage
Sex of caregivers		
Male	36	23.2
Female	119	76.8
Caregivers’ age (M = 41.60; SD = 10.38)		
18–40	82	52.9
41–60	64	41.3
60 +	9	5.8
Caregivers’ education		
No education	11	7.1
Primary education	62	40.0
Secondary education	35	22.6
Tertiary education	47	30.3
Caregivers’ relationship with child		
Biological parent	85	54.8
Extended family	48	31.0
Guardian	22	14.2
Disclosure of status		
Disclosed	52	33.5
Non-disclosed	103	66.5
Child’s age (M = 9.55; SD = 2.72)		
6–10	95	61.3
11–15	60	38.7
Sex of child		
Male	83	53.5
Female	72	46.5
Child’s education		
Primary education	144	92.9
Secondary education	11	7.1

### Relationship among disclosure, length of disclosure, and health outcomes

The relationship among the study variables- disclosure, length of disclosure, medication adherence, psychological wellbeing of children, caregiver burden, and psychological health of caregivers are presented in Table 2. Disclosure of HIV status showed a negative and strong significant relationship with the length of disclosure [*r* (155) =  − 0.76, *p* < 0.01], moderate and negative relationship with medication adherence among the children [*r* (155) = − 0.53, *p* < 0.01], and psychological wellbeing of children [*r* (155) =  − 0.63, *p* < 0.01], but showed a weak and positive relationship with caregiver burden [*r* (143) = 0.17, *p* < 0.05]. Length of disclosure was found to have a significant positive and moderate relationship with medication adherence [*r* (155) = 0.48, *p* < 0.01] and psychological wellbeing of caregivers [*r* (154) = 0.58, *p* < 0.01]. Additionally, a significant moderate and positive relationship was observed between medication adherence and psychological wellbeing in children living with HIV [*r* (154) = 0.60, *p* < 0.01], while caregiver burden was positively associated with psychological health of caregivers [*r* (135) = 0.61, *p* = 0.01].

**Table 2:** Correlation matrix of study variables

		1	2	3	4	5
1	Disclosure of Child’s HIV Status (*yes* = *1*)	1				
2	Length of Disclosure	− .76**	1			
3	MACHIV	− .53**	.48**	1		
4	PWCHIV	− .63**	.58**	.60**	1	
5	CB	.17*	− .13	− .06	.02	1
6	PHC	.08	− .01	− .07	.01	.61**

*MACHIV* Medication Adherence of Children living with HIV, *PWCHIV* Psychological Wellbeing of Children living with HIV, *CB* Caregiver Burden, *PHC* Psychological Health of Caregivers

*p < .05, **p < .01

### Factors associated with disclosure of HIV status

Results from the regression analysis are presented in Table 3. The first model in disclosure of status controlling for demographic variables of the child and caregiver was significant *F* (3, 130) = 20.65, *p* < 0.001; *R*^*2*^ = 0.32, with child’s age (*β* = − 0.41, *p* < 0.01) and child’s educational level (*β* = − 0.27, *p* < 0.01) making a statistically significant contribution while caregivers’ educational level (*β* = 0.08, *p* = 0.27) was not a significant contributor. The last model which was of interest to the researchers was significant, Δ*R*^2^ = 0.16, *F* (7, 126) = 16.60, *p* < 0.001. After controlling for demographics variables, medication adherence (*β* = − 0.16, *p* < 05), psychological wellbeing of children living with HIV (*β* = -− 0.35, *p* < 0.001) and caregiver burden (*β* = 0.17, *p* = 05) significantly predicted disclosure of HIV status. These factors accounted for a unique variance in disclosure (*p* < 0.001, Adj. *R*^2^ = 0.45). This means that holding other variables constant, the chance of disclosure increased 16 times due to either adherence concerns, a child’s psychological wellbeing concerns or burden of care and this explains 45% of the variance in disclosure. The psychological health of caregivers was however not associated with disclosure of HIV status (*β* = − 0.07, *p* = 0.41).

**Table 3:** Hierarchical regression indicating factors that influence disclosure of HIV status

Model		*B*	(SE *B*)	*Β*	*R*^*2*^	Δ*R*^*2*^	*F*	*p*
1					.32		20.65	< .01
	(Constant)	2.82	.16					
	Child’s Age	− .07	.01	− .41**				
	Child’s level of education	− .47	.14	− .27**				
	Caregiver's level of education	.04	.03	.08				
2					.48	.16	16.60	< .01
	(Constant)	3.23	.22					
	Child’s Age	− .04	.01	− .22				
	Child’s level of education	− .26	.13	− .15				
	Caregiver's level of education	.03	.03	.07				
	MACHIV	− .04	.02	− .16*				
	PWCHIV	− .02	.01	− .35**				
	CB	.01	.00	.17*				
	PHC	− .00	.00	-.07				

*MACHIV* Medication Adherence of Children living with HIV, *PWCHIV* Psychological Wellbeing of Children living with HIV; *CB* Caregiver Burden, *PHC* Psychological Health of Caregivers. *B*  Unstandardized coefficient B; *SE B* = Standard error of B; *β * Standardized coefficients beta

*p < .05, **p < .01

## Discussion

This study examined the factors influencing disclosure of HIV status of children by their caregivers in Ghana. Our results revealed several important observations. First, approximately one-third of the children had full disclosure of their HIV status. Second, disclosure of HIV status was significantly related to medication adherence, psychological wellbeing of children, the burden of caregiving, and the length of disclosure. Third, children’s age and level of education were the only demographic variables that significantly predicted disclosure of HIV status. Finally, after controlling for all demographic variables medication adherence, psychological health, and burden of caregiving were found to be significant predictors of disclosure of status in children living with HIV.

Findings showed that the rate of full disclosure of status to children living with HIV is still low in Ghana. Although this rate of disclosure is considered to be low, it is slightly higher compared to those reported in Kenya [31], the Dominican Republic [29], Zimbabwe [32], and earlier figures reported in Ghana [15]. The low rate of disclosure of HIV status could be attributed to the fact that the majority of the children who participated in this study were younger, and it has been established that older children and adolescents are more likely to have their HIV status disclosed to them than younger children [15, 33, 34]. It is plausible that older children and adolescents living with HIV have adequate knowledge about HIV, have become responsible for self-care and so disclosure of status is necessary for adherence to ART. In Ghana, it has been established that adolescents living with HIV who were knowledgeable about HIV/AIDS were more likely to have their HIV status disclosed to them [35, 36].

Disclosure of HIV status was significantly associated with medication adherence in children living with HIV. This means the more parents do not disclose children’s status, the less the children adhered to their medication intake. Previous studies have confirmed this association [15, 28, 33, 34]. For instance, in Peru, Baker and colleagues [33] found that caregivers and health care professionals’ frustrations about non-adherence to medication were compelled to disclose the status of their children living with HIV. This could be because most caregivers do not explain to children living with HIV/AIDS why they are on medication daily and without these explanations, it is difficult for these infected children to see the need to continue taking medications and therefore they may miss doses, take them irregularly, or even stop taking the medications altogether. Thus, in a bid to ensure medication adherence, disclosure has to be made.

Findings also showed that psychological wellbeing significantly influenced the disclosure of status in children living with HIV, supporting earlier studies [13, 14]. For example, Nichols and colleagues [14] found in a systematic review that disclosure resulted in improved psychological wellbeing in children living with HIV who had their status disclosed to them than children living with HIV whose status had not been disclosed to them. Research has indicated that lack of disclosure creates tension and suspicion in families as there are no open discussions in the family setting about the child’s condition [7, 11, 12]. This culture of silence can make children living with HIV curious and inquisitive about their condition and this can result in behavioural problems and destroy familial peace and cohesion if questions and concerns that children have are not addressed. As a way to prevent these, disclosure is made to these children to ensure their minds are clear on the need for medication. Disclosure also clears tensions and the curiosity that these children have about their condition.

Disclosure of status in children living with HIV was associated with elevated levels of caregiver burden. This finding corroborates findings by Gyamfi et al. [13] that disclosure is likely to reduce burden in caregivers as children adhere to medication and play an active part in managing their conditions, such as reminding parents of allergies, the timing of medications and timing of reviews. A possible explanation for the present finding is that disclosure is likely to make children understand their condition and take more responsibility for their health and wellbeing, thereby reducing the burden in caregivers monitoring medication adherence and ensuring frequent hospital visits and check-up. Research has indicated that the welfare of children living with HIV greatly depends on their primary caregivers who aid in providing most health needs and also provide support for children’s social and emotional development [37]. This can create a severe burden of care whether physical, psychological, emotional, social, or financial for caregivers [18, 20].

The psychological wellbeing of caregivers in the present study was however not a significant factor that influenced disclosure of status. A possible explanation is that majority of caregivers may still entertain fears that children will divulge the news of their status to others. Also, the fear of stigmatization coupled with the many financial and social challenges that most caregivers encounter could probably explain why psychological health did not influence the disclosure of status to children caregivers. This is because these challenges are consistently raised by caregivers of children living with HIV regardless of whether they have disclosed the status of their children to them or not [21–20].

In summary, the need for optimum adherence and improved psychological wellbeing of the children living with HIV, as well as a reduced burden of caregiving are some of the factors that predicted disclosure among caregivers of children living with HIV in Ghana. As such guidelines towards encouraging the disclosure of status to children living with HIV in Ghana should be tailored with these factors in mind, to produce the desired health outcomes in these children and their caregivers.

### Limitations and recommendations for future research

There are limitations of this study and hence the need to interpret findings in the light of these limitations. First, the study used a cross-sectional design, which only allows associations to be drawn between predictor variables and the outcome variables. Therefore, causation cannot be inferred from the results obtained. Second, the use of self-report measures in assessing both predictor and outcome variables is sometimes characterized by some predispositions or biases, giving social desirable responses, set or random responses [38], and this could have been the case for this study. Third, the use of clinical tests such as viral load test and pill count were not utilized. Viral load tests could have provided more accurate information about the children’s HIV clinical outcomes (CD4 count) and pill count could have complimented the measure of adherence to medication. The above notwithstanding, this study contributes to understanding the factors that influence the disclosure of status by their caregivers of children living with HIV as well as the influence of disclosure of HIV status. Future studies can adopt a longitudinal study design to examine the factors that influence the disclosure of status in children living with HIV over time. Also, future studies can include multiple measures other than self-report measures to counter some of the biases that self-report measures are prone to. For example, medication adherence could be measured using both self-report measures as well as clinical tests such as CD4 tests and viral load tests as well as pill count or pharmacy count.

### Implications for clinical care and practice

The findings of the study have some implications for practice and clinical care. There is the need for the Ministry of Health and the Ghana Health Service to formulate and implement policies to encourage the disclosure of status to children living with HIV. In developing this disclosure framework, there is also the need to consider some contextual factors that prohibit and enhance disclosure as established by research. For instance, age should be one of the factors to be considered in developing a national disclosure framework. Besides, the focus should be on the need for optimal medication adherence, improved psychological wellbeing, and the reduction of the burden of care, as these factors were found to significantly influence the disclosure of status among caregivers of children living with HIV/AIDS.

## Conclusion

This study revealed that status disclosure to children living with HIV is still low in Ghana hence there is the need to educate caregivers on the importance of disclosure to children. Similarly, findings showed the need for medication adherence, psychological wellbeing, and reduction of the burden of care significantly influence the disclosure of status in children living with HIV by their caregivers. Disclosure therefore should be encouraged among children living with HIV with special focus on medication adherence, psychological wellbeing.

## Data Availability

The data for this study are not publicly available because the data forms part of a bigger study from which other manuscripts are being written. Thus, all data and supporting materials will be made available from the corresponding author (on agreement with the co-authors) on reasonable request.

## Abbreviations

AIDS: Acquired immunodeficiency syndrome
ART: Antiretroviral therapy
HIV: Human Immunodeficiency Virus
SD: Standard deviation
SPSS: Statistical Package for the Social Sciences
